# Development of 4D-Printed Arterial Stents Utilizing Bioinspired Architected Auxetic Materials

**DOI:** 10.3390/biomimetics10020078

**Published:** 2025-01-26

**Authors:** Nikolaos Kladovasilakis, Ioannis Filippos Kyriakidis, Emmanouil K. Tzimtzimis, Eleftheria Maria Pechlivani, Konstantinos Tsongas, Dimitrios Tzetzis

**Affiliations:** 1 Digital Manufacturing and Materials Characterization Laboratory, School of Science and Technology, International Hellenic University, 14th km Thessaloniki-Moudania, 57001 Thessaloniki, Greece; n.kladovasilakis@ihu.edu.gr (N.K.); m.tzimtzimis@ihu.edu.gr (E.K.T.); 2 Centre for Research and Technology Hellas, Information Technologies Institute (CERTH/ITI), 57001 Thessaloniki, Greece; giankyri@iem.ihu.gr (I.F.K.); riapechl@iti.gr (E.M.P.); 3 Advanced Materials and Manufacturing Technologies Laboratory, Department of Industrial Engineering and Management, School of Engineering, International Hellenic University, 57001 Thessaloniki, Greece

**Keywords:** auxetic materials, topology optimization, biodegradable materials, biomedical applications, additive manufacturing, finite element analysis

## Abstract

The convergence of 3D printing and auxetic materials is paving the way for a new era of adaptive structures. Auxetic materials, known for their unique mechanical properties, such as a negative Poisson’s ratio, can be integrated into 3D-printed objects to enable them to morph or deform in a controlled manner, leading to the creation of 4D-printed structures. Since the first introduction of 4D printing, scientific interest has spiked in exploring its potential implementation in a wide range of applications, from deployable structures for space exploration to shape-adaptive biomechanical implants. In this context, the current paper aimed to develop 4D-printed arterial stents utilizing bioinspired architected auxetic materials made from biocompatible and biodegradable polymeric material. Specifically, three different auxetic materials were experimentally examined at different relative densities, under tensile and compression testing, to determine their mechanical behavior. Based on the extracted experimental data, non-linear hyperelastic finite element material models were developed in order to simulate the insertion of the stent into a catheter and its deployment in the aorta. The results demonstrated that among the three examined structures, the ‘square mode 3’ structure revealed the best performance in terms of strength, at the same time offering the necessary compressibility (diameter reduction) to allow insertion into a typical catheter for stent procedures.

## 1. Introduction

Additive manufacturing (AM), known as 3D printing, has gained increased attention in recent years due to the ability to rapidly fabricate complex designs, the design flexibility given by the quick customization and the time saved from the digital 3D model to the final structure, along with the minimal waste production during the process [1,2]. This design flexibility allows the insertion of AM into numerous fields of study for automotive, aerospace and biomedical applications. Especially in the biomedical field, where personalized designs are demanded, AM is a go-to technique for the rapid development and production of patient-specific products with high dimensional accuracy and precise detail. Furthermore, advanced 3D printing technologies, in conjunction with state-of-the-art materials, have the potential to revolutionize the fabrication of biomedical products. These technologies enable the development of structures with unique physico-chemical and mechanical properties, leading to superior performance in practical applications. A comprehensive review by Li et al. [3] highlights recent progress in this field, focusing particularly on piezoelectric composites integrated within advanced lattice structures. These composites exhibit intelligent and functional diversification, effectively addressing the complex demands of practical biomedical applications. Moreover, the advanced designs that are possible with AM techniques are offering increased biomimicry behavior, facilitating interference with the human body and enhancing their efficiency. Therefore, advanced biomaterials with controlled porosity, mechanical properties and biocompatibility can be efficiently produced, paving the way for next-generation biomedical enhancements.

Biodegradable polymers, such as polylactic acid (PLA) and polycaprolactone (PCL), have shown great biocompatibility with the human body and have been widely used for scaffolding in tissue engineering [4,5]. Especially, PCL is one of the most well-known biopolymers for tissue engineering, already approved by the FDA, and presents numerous advantages for tissue engineering applications due to its relatively low melting point (59–64 °C) and glass transition temperature (around 60 °C) allowing easy surface modification, ease of synthesis and high drug permeability. It also presents good hydrophobicity, providing good cell adhesion, and the degradation rate is slow enough to allow the full nutrient substances or drugs to be released into the human body, making it adequate for scaffolding for such applications [6,7,8,9].

Nowadays, biodegradable arterial stents, also known as bioresorbable vascular stents (BVSs), are currently used in certain medical applications where the artery needs to heal from a disease, such as coronary artery disease (CAD), or in younger patients where permanent metallic implants are not ideal due to growth or the need for future surgeries [10,11]. Conventional biodegradable arterial stents are deployed via a minimally invasive angioplasty procedure [12]. A catheter with a stent-mounted balloon is inserted through an artery in the wrist or groin and guided to the blockage using X-ray imaging. The balloon inflates to expand the stent, opening the artery and restoring blood flow. The balloon is then deflated and removed, leaving the stent to support the artery while it gradually dissolves over time. However, balloon use carries certain risks, such as arterial wall damage, plaque embolization, and restenosis [13]. Therefore, in recent years, shape memory polymers (SMPs) have been proposed, enabling self-expanding stents that deploy autonomously upon release from the catheter, reducing reliance on balloons and potentially lowering complication rates.

The introduction of shape memory polymers (SMPs) has allowed the extension of 3D printing to 4D printing for the construction of smart materials that can reshape and reform due to their abilities to respond to external stimuli, such as external force, chemical changes, temperature changes and electromagnetic changes. Many studies have focused on creating a benchmark describing the optimal shape memory properties for potential scaffolding made out of PLA and PCL matrices, with the results showing that PLA/PCL blends presented a good shape fixity ratio of around 91% and a shape recovery ratio of 90%. It was also shown that PCL scaffolds manufactured by fused filament fabrication (FFF) presented the attainability of their mechanical properties and cell adhesion for a repetitive number of heating cycles for the production of two-way shape memory materials [14,15,16,17,18,19,20,21,22,23]. Peng et al. [24] focused on the effects of the manufacturing process on the shape memory abilities of the final structures and found out that scaffolds extracted by AM processes presented better stability than those coming from other conventional manufacturing techniques such as compression molding, while Zhu et al. [25] and Li et al. [26] addressed the optimal printing conditions for the best shape memory performance of PCL matrices, finding a wide range of conditions that presented a minimal effect on the final properties of the structure. Moreover, 4D-printed structures with a negative Poisson’s ratio present increased energy absorption and flexibility due to their ability to expand and shrink laterally. Topology optimization allows the introduction of structures with tailored properties and great strength-to-weight ratios, allowing the manufacturing of medical enhancements with great compatibility with the human body. Combining the shape memory properties of PCL with topology optimization utilizing auxetic materials can allow the development and fabrication of novel stents for arterial stenosis that are self-deployed and can conform better to the arterial walls and reduce the risk of reshrinking of the artery [27,28,29,30,31].

**Figure 1:**
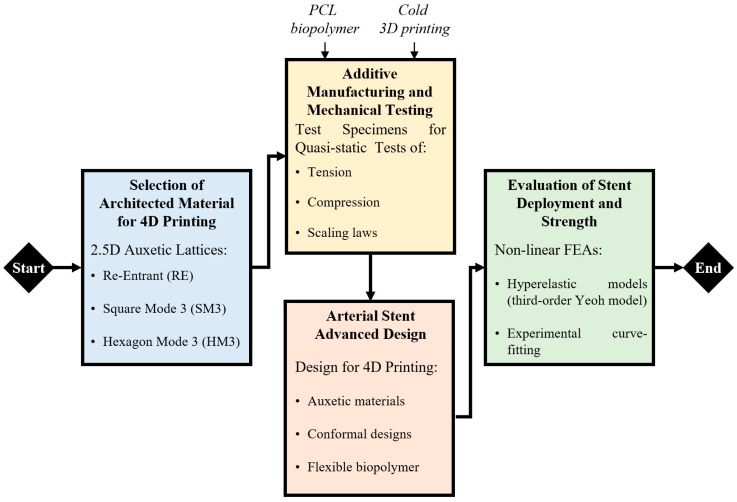
Flowchart of this research.

This study introduces a novel approach for the development of a self-deployable, bioresorbable and robust arterial stent by exploiting the 4D printing framework with advanced auxetic materials. Specifically, three innovative auxetic materials with a negative Poisson’s ratio were designed, fabricated with the aid of FFF 3D printing technology, and examined in order to find the most suitable auxetic material for arterial stent application. The construction material was PCL, and three different types of auxetic architected materials were applied in the constructed specimens. The specimens were then subjected to uniaxial tensile and compression testing to assess the main mechanical properties and observe the structures morphing under loads. The results were used to develop non-linear finite element material models, where arterial stent designs can be numerically examined. Then, prototypes of the developed stent were designed, and a computational investigation of their behavior was accurately simulated with the aid of finite element analysis (FEA). In Figure 1, a brief flowchart of this study is illustrated. The first step involved designing the selected architected auxetic materials and defining the methodology for the tensile and compression testing, as well as the FEAs. Finally, the optimal arterial stent designs were extracted and in-depth numerical analyses were performed in order to simulate catheter extraction and deployment of the stent.

## 2. Materials and Methods

### 2.1. Design of Auxetic Materials and Arterial Stent

**Figure 2:**
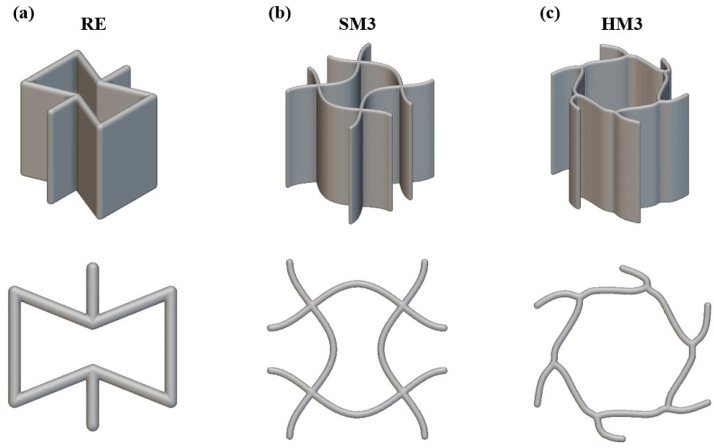
Three-dimensional model for the unit cells of (**a**) RE; (**b**) SM3 and (**c**) HM3.

The first step was the selection and design of the architected auxetic materials. For an arterial stent application, the selected structures should have a 2.5D configuration in order to be integrated conformally into the conventional stent geometry [32]. As the first auxetic material, the re-entrant (RE) hexagonal architected material was selected. The RE is one of the most well-known and commonly used auxetic materials, as when it undergoes tension loads, the diagonal element rotates, leading to the lateral extension of the overall structure and resulting in the auxetic effect with a remarkable negative Poisson’s ratio of around −0.4 [33]. The other two auxetic materials, namely the square eigenmode 3 (SM3) and the hexagonal eigenmode 3 (HM3), were selected based on their potential in 2D auxetic applications. More specifically, these two auxetic materials achieve negative Poisson’s ratios of −0.3 and −0.2, respectively [34]. It is worth noting that these two structures emerged from the employment of the computational electrodynamics modeling technique eigenmode expansion [35] and this is the first time that they are examined in a 2.5D configuration. Regarding the design of this auxetic material, first, the unit cells were designed based on the 2D geometry of the surface models in SolidWorks™ 2023 design software (Systèmes SolidWorks Corp., Waltham, MA, USA). Then, these 3D surface models were inserted in .STEP format into nTopology software, where they were defined as fully parametrized custom unit cells. Via this process, all the available design tools of the nTopology platform, such as conformal design, unit cell manipulation, etc., can be employed on the developed auxetic materials. Figure 2 shows the 3D model of each developed unit cell, along with the corresponding cross-section.

**Figure 3:**
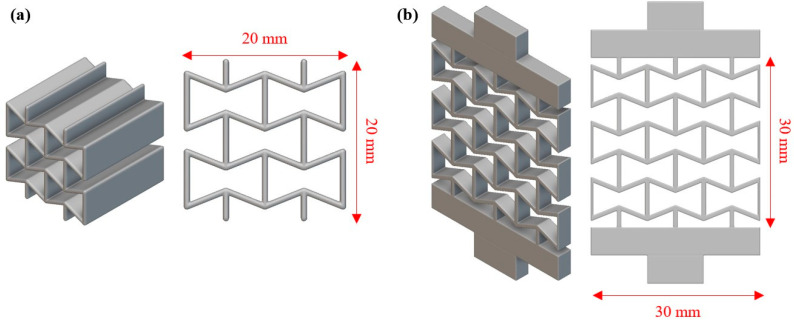
Indicative images of the configuration for (**a**) compression specimens, and (**b**) tensile specimens.

Having the designs of the unit cells for the selected auxetic materials, the next step was the design of the necessary test specimens. For the compression testing, a cubic 2 × 2 configuration with a length of unit cell at 10 mm was chosen, resulting in specimens of 20 × 20 × 20 mm^3^. On the other hand, for tensile testing, a 3 × 3 configuration was employed due to the size of the testing machine’s grippers. Again, the length of the unit cell was set at 10 mm while the thickness was set at 3 mm. Also, special configurations on the upper and lower sides of each specimen were designed in order to facilitate positioning in the testing machine. Figure 3 illustrates indicative images of the compression and tensile test specimens.

In order to extract the overall mechanical behavior of an architected material, it is necessary to quantify the scaling law that each one obeys. Therefore, the mechanical properties of each examined architected material should be evaluated in terms of at least three relative densities. For this reason, in the context of this study, specimens of three different relative densities were designed and developed by adjusting each time the value of the wall thickness. Table 1 lists the corresponding design values for each selected relative density. It is worth noting that the wall thicknesses were selected as multiples of 0.4 mm based on the discretization and the accuracy of the employed 3D printing process (nozzle diameter at 0.4 mm).

The final step was the design of the arterial stents. As a use case scenario, the placement of the developed stent was in an aorta, and more specifically, in the section of the descending and abdominal aorta. In these sections, the nominal internal diameter is around 20 mm [36]; therefore, the developed stents were designed to have an external diameter of 20 mm. Moreover, the thickness of the stents was set at 3 mm in order to provide the necessary structural integrity for the support of the aorta. In addition, the length of aortic stents can vary from 50 to 200 mm depending on the extent of the problem [37]. In the examined case, aortic stents of 50 mm were designed in order to facilitate the computational process and sufficiently prove the examined concept. Regarding the conformalization methodology, to achieve sufficient conformality of the applied auxetic materials on the stent geometry, the internal and external cylindrical surfaces of the solid stent were selected as boundary conditions for the lattice structure. These surfaces define the variable curvature pattern from the internal to the external diameter. The final step in the conformal design process involved defining the number of inscribed unit cells of each auxetic material within the cylindrical arc, namely 4 unit cells were applied. Additionally, the thickness of the lattice structures was set at 3 mm, corresponding to the difference between the internal and external diameters of the stent’s cylinder.

### 2.2. Additive Manufacturing

In the additive manufacturing process, the fused filament fabrication (FFF) technique was employed using a Prusa i3 MK3S+ 3D printer (Prague, Czech Republic). The feedstock material used was eSun Natural eMate PCL filament, a highly biodegradable material exhibiting more than 10% weight loss after three months [38,39]. The 3D printing was conducted at room temperature with minimal moisture content. No support structures were required due to the self-supporting design of the architected materials. As suggested in the literature [40], Table 2 presents the key printing parameters used in the manufacturing process, alongside the basic material properties provided by the manufacturer. Importantly, the orientation of the printed structure was chosen to ensure that the layers were perpendicular to the compression loading direction and parallel to the tensile loading direction, optimizing the mechanical performance [41,42]. Finally, at least three specimens were tested for each experiment to maintain the reliability of the experimental procedure.

### 2.3. Mechanical Testing

The test specimens that were described in Section 2.1 underwent tensile and compression tests in order to extract the mechanical behavior of the examined auxetic materials. Firstly, the 3D-printed construction material’s properties were provided by our previous study [40] and are listed in Table 3.

The next step was the evaluation of the overall mechanical behavior of the examined auxetic material. Auxetic materials are classified as architected materials, and according to the existing literature [43,44], all architected materials obey specific scaling laws that quantify the size effect of the relative density. In detail, the size effect is a mechanism that reveals how the mechanical behavior is compromised by the variation of the relative density and it mainly affects architected materials with relative densities below 50% [45]. The equation below presents the aforementioned scaling law, where Φ_solid_ is the mechanical property of the construction material, Φ_lattice_ is the effective mechanical property for the architected material, and G and n are constants so that their values depend on the construction material and the applied architected material.

(1) $$\frac{\Phi_{lattice}}{\Phi_{solid}}=G\cdot {\left(\bar{\rho }\right)}^{n}$$

For this reason, specimens with three different relative densities were designed, fabricated and tested. Then, the test results were used in order to curve-fit the observed mechanical behavior with the scaling law equation. Through this process, an overall image of each structure’s mechanical behavior was extracted, providing a computation tool for approximately quantifying the examined properties for a relative density range of 5–50% with high accuracy and reliability. Finally, it is worth mentioning that the uniaxial quasi-static compressive and tensile tests were conducted at least three times with a universal testing machine, Testometric-M500-50AT (Rochdale, UK), equipped with a 50 kN load cell and the quasi-static strain was selected as 5 mm/min [46].

### 2.4. Finite Element Model

After the extraction of the mechanical behavior for each examined auxetic material, the next step was the development of a finite element material model. For this purpose, the static module of the ANSYS™ (ANSYS, Inc., Canonsburg, PA, USA) simulation platform was utilized. These material models were developed based on the experimental behavior of the auxetic materials. Through the testing process, it was observed that the PCL architected materials exhibited non-linear hyperelastic behavior, especially at the low relative densities (<30%). This non-linear hyperelastic behavior, as observed in the stress–strain diagram, exhibits two inflection points where the curvature changes. To capture this behavior, a 3rd-order material model was required. Previous research [43,44,47] demonstrated that the 3rd-order Yeoh model yielded the lowest error residual in simulating the mechanical behavior of architected materials. For this reason, the non-linear hyperelastic 3rd-order Yeoh material model was selected as it is able to simulate accurately and reliably the complex mechanical behavior of architected materials. It is worth noting that the 3rd-order Yeoh material model employs the formulation for the strain energy density (W) and is evaluated by the following equation:

(2) $$W\left(I_{1}\right)=\sum_{i=1}^{3}C_{i}{\left(I_{1}-3\right)}^{i}\;\mathrm{with}\;I_{1}=tr\left(C\right)$$

where I_1_ the first strain invariant and C_i_ are the material model constants that were calculated properly to match the non-linear sections of the stress–strain diagrams for the examined auxetic materials. Having selected and set the material model, then, for the simulation for each auxetic material, a curve-fitting process was conducted, matching the experimental data with the constants of the Yeoh model equation. More specifically, in the curve-fitting process, the uniaxial experimental data acquired from the compression tests on each auxetic material were used to determine the material constants (C_i_) of the 3rd-order Yeoh model. Initially, uniaxial stress–strain values were gathered through experimental testing. Subsequently, the ANSYS software (Ansys Academic Research Mechanical, Release 24.1) was employed to determine the C_i_ values by solving the Yeoh model equation iteratively. The software minimizes the error between the predicted stress–strain curve from the model and the experimental data, ensuring an accurate representation of the material’s mechanical behavior. Regarding the applied mesh for each developed stent, mesh sensitivity analyses were conducted for each developed stent to ensure mesh-independent results, with a focus on the equivalent stresses. Based on these analyses, the element size range was determined to be between 0.2 mm and 0.4 mm, enabling the accurate representation of the stents’ complex lattice geometries. This resulted in tetrahedral meshes comprising approximately 200,000–230,000 elements, depending on the specific geometry. For instance, the RE geometry mesh consisted of 445,853 nodes and 217,088 elements, the HM3 geometry included 406,770 nodes and 210,598 elements, and the SM3 geometry was composed of 482,424 nodes and 225,316 elements. The developed meshes employed the SOLID187 element type, a tetrahedral solid element with three degrees of freedom per node, ensuring accurate modeling of the stents’ mechanical behavior. The objective of the FEA was to verify that a compression of 70% of strain, i.e., a diameter decrease from 20 mm to around 6 mm, is possible without compromising the material integrity (elastic deformation) in order to re-deploy in the initial size when uncompressed. It is crucial to mention that for the typical placement of a descending aorta stent, a 18F to 26F catheter is used (i.e., internal diameter ranging from 6 mm to 8.3 mm) [48]. Therefore, as the loading conditions in the conducted FEAs, the radial displacement was set up to 70% of the strain. Regarding the fixation of the stent, rotations and movements in the other two dimensions of the cylindrical coordination system were not allowed. The loads were applied in ten distinct steps in order to facilitate the convergence of the solver.

## 3. Results

### 3.1. Fabrication and Mechanical Testing Results

**Figure 4:**
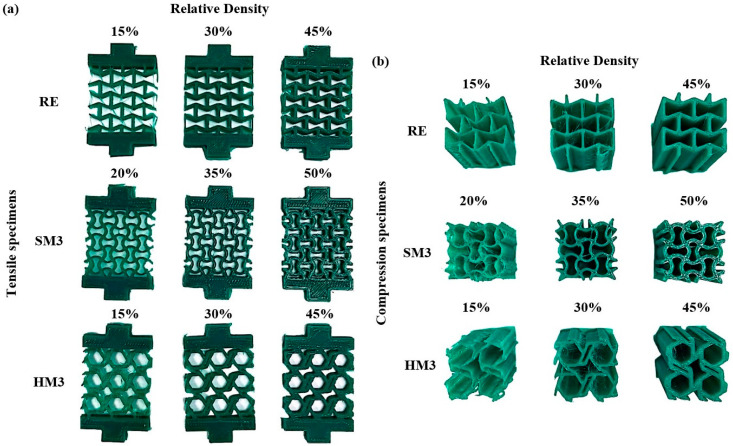
Indicative images of the (**a**) tensile and (**b**) compression test specimens for all the examined relative densities.

In order to extract the mechanical behavior of the examined auxetic materials, tensile and compression specimens at three different relative densities were designed and fabricated via FFF 3D printing utilizing PCL as the construction material. Figure 4 portrays the 3D-printed test specimens for each auxetic structure, along with their as-designed relative densities. The 3D-printed specimens were manufactured without severe defects following sufficiently the as-designed geometry, especially for relative densities above 20%. It is worth noting that for relative densities of 15%, the structures were fragile, increasing the risk of deformation during the removal from the build platform. In addition, the as-built relative densities revealed negligible deviations from the as-designed relative densities ranging ±2%.

After the fabrication of the test specimens, the next step was the conducting of mechanical testing experiments. During these experimental tests, the main effective mechanical properties, namely the elastic modulus, yield stress, ultimate tensile strength (UTS) and compressive strength, for each auxetic material were evaluated. Table 4 lists the values of the mechanical properties for each auxetic material at all the examined relative densities. Based on the experimental data in Table 4, structure SM3 revealed the stiffest behavior along with the highest strength, followed by structures RE and HM3. However, as the relative density of the structures increases, the performance of the HM3 structure improves, both in terms of the stiffness and in terms of the strength, especially in tension loading, which surpasses the SM3 structures. Overall, all the examined auxetic materials showed elastomeric behavior and achieved an elastic modulus (elasticity) and resilience comparable with rubber materials such as neoprene and natural rubber [49]. In addition, the strain exceeded 200% for tensile testing without compromising the structural integrity of the material. These observations led to the result that PCL auxetic materials are suitable candidates for 4D-printed products using a stimulus external force to modify their shape and release it when the product reaches its final destination to recover the as-designed geometry.

Utilizing these experimental data, it is possible to extract the scaling laws for each examined auxetic material, providing a computational tool to assess the main effective mechanical properties for a wide range of relative densities. The scaling laws were evaluated by quantifying the corresponding C_Φ_ and n variables and curve-fitting them with the experimental results. It is worth noting that Φ represents the corresponding property. Table 5 lists the C and n values for each auxetic material and for each examined mechanical property.

**Figure 5:**
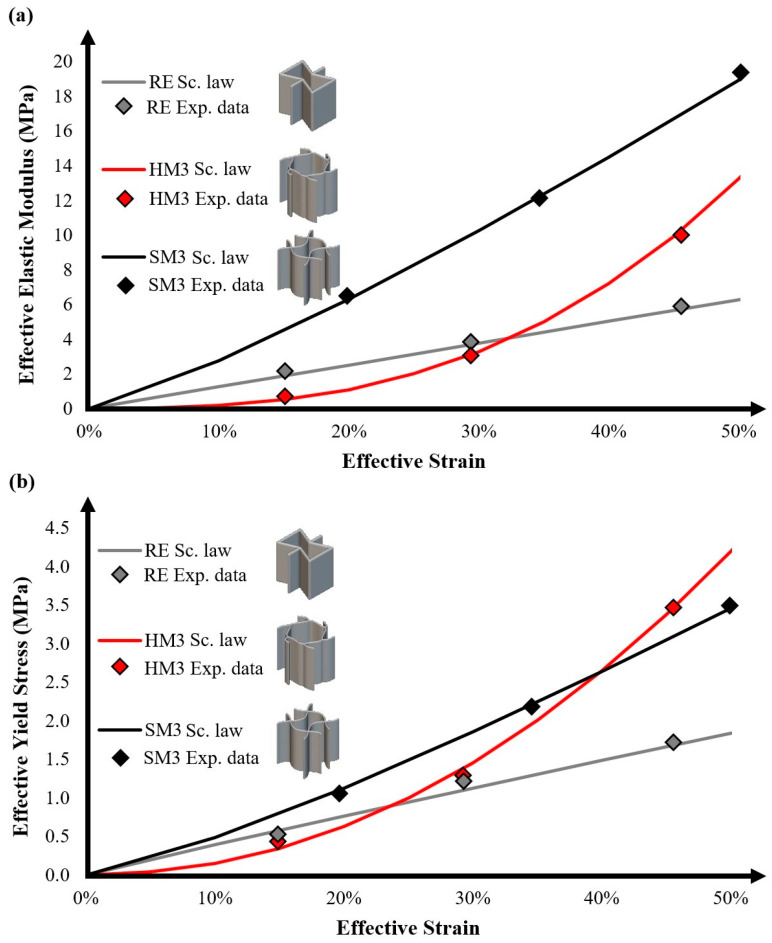
Quantitative diagrams for each auxetic structure of (**a**) the effective elastic modulus and (**b**) the effective yield stress.

Scaling laws are indicators of the mechanical behavior of architected materials, especially the value of n for the effective elastic modulus as it indicates if the structure reveals stretching-dominated (n ≈ 1) or bending-dominated behavior (n ≥ 2). According to the extracted n values for the effective elastic modulus for the examined auxetic materials, SM3 revealed clear stretching-dominated behavior, with an almost linear relation between the elastic modulus and the relative density. This means that SM3 has high connectivity among the struts of the lattices, resulting in sufficient strength and more stiff behavior. Similar conclusions can be extracted for RE as it has milder stretching-dominated behavior. Finally, HM3 revealed strong bending-dominated behavior, which leads to increased bending of its structural elements during loading. The bending-dominated behavior also resulted in poor mechanical performance at low relative densities (<20%), which is corrected as the relative density increases (>30%). Figure 5a depicts the quantitative diagram of the scaling laws of the examined auxetic materials regarding the effective elastic modulus.

**Figure 6:**
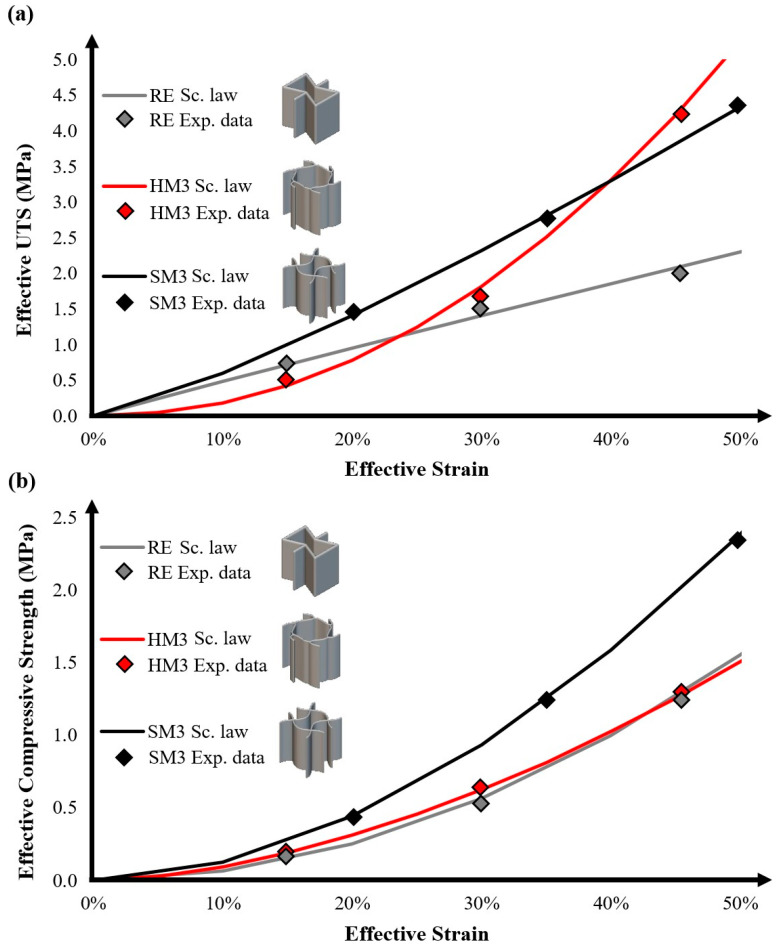
Quantitative diagrams for each auxetic structure of (**a**) the effective UTS and (**b**) the compressive strength.

Moreover, Figure 5b shows the corresponding diagram for the effective yield stress and Figure 6a,b illustrate the quantitative diagrams for the effective UTS and effective compressive strength, respectively. According to these diagrams, the curve-fitting process has been performed sufficiently, with only minor deviations from the experimental data, resulting in a correlation coefficient R above 0.98. In detail, the mean absolute percentage errors (MAPEs) between the scaling laws and the experimental data were found to be below 10%, demonstrating the reliability of the proposed models; these errors can be further minimized when low relative densities (<15%), which are associated with poor printability using FFF 3D printing technology, are excluded. Regarding the yield stress and the UTS, the auxetic structures followed identical trends. On the other hand, for the compressive loading, the examined auxetic materials revealed an exponential response, showing the elasticity and deformability of the structures, characteristics that are essential for arterial stent application due to the compression of the stent inside the catheter for proper placement in the artery.

### 3.2. Arterial Stent Designs

**Figure 7:**
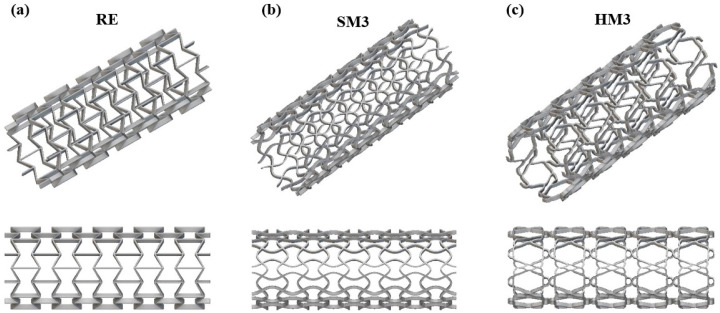
Three-dimensional models of the developed arterial stents for the (**a**) RE, (**b**) SM3 and (**c**) HM3 auxetic structures.

After extracting the mechanical behavior of each selected architected auxetic material, the next step involved integrating them into a stent-like geometry and evaluating their functionality and compressibility through computational analyses. Figure 7 shows the 3D models of the aortic stents for the three selected auxetic materials. Regarding the design of the stent, a conformal application of the auxetic material was employed, meaning that the unit cell of each material followed the external geometry of the stent in order to have a cylindrical shape. Furthermore, each stent possesses eight unit cells in the radial direction of 10 mm length with 3 mm radial thickness and 50 mm indicative length. In addition, the relative density was set at 30% in order to obtain sufficient structural integrity and achieve better manufacturability. For this purpose, the wall thicknesses were selected as 0.8 mm for RE and HM3 and 0.6 mm for SM3. It is worth mentioning that the length of this stent design is fully scalable depending on the patient-specific condition.

### 3.3. Finite Element Analysis

The final step in this study was the computation examination of the developed aortic stents utilizing finite element analyses. The purpose of the FEAs was to examine the stents during the placement phase, where they are located inside the catheter. During this phase, the stent diameter needs to be reduced by almost 70% of the strain in order to fit inside a 18F catheter tube (worst case), i.e., the diameter should be reduced from 20 mm (initial) to around 6 mm (inside the catheter). This reduction is achieved by applying radial compressive pressure and forcing the elements of the structure to bend. Due to the utilization of the elastic biopolymer PCL and the integration with novel architected auxetic materials, this deformation can be achieved without compromising the structural integrity of the stent. In this subsection, the FEAs verify the compressibility of the designed stents and extract the most suitable candidate structure for further research in the future.

In order to perform realistic and reliable FEAs, hyperelastic material models were employed based on the acquired experimental data for each examined auxetic structure. A third-order Yeoh hyperelastic material model was used; therefore, three distinct material model constants should be evaluated, i.e., C_1_, C_2_ and C_3_. For the accurate evaluation of these constants, at least five points of uniaxial experimental data were utilized. Table 6 lists the values of these constants for each examined auxetic material.

**Figure 8:**
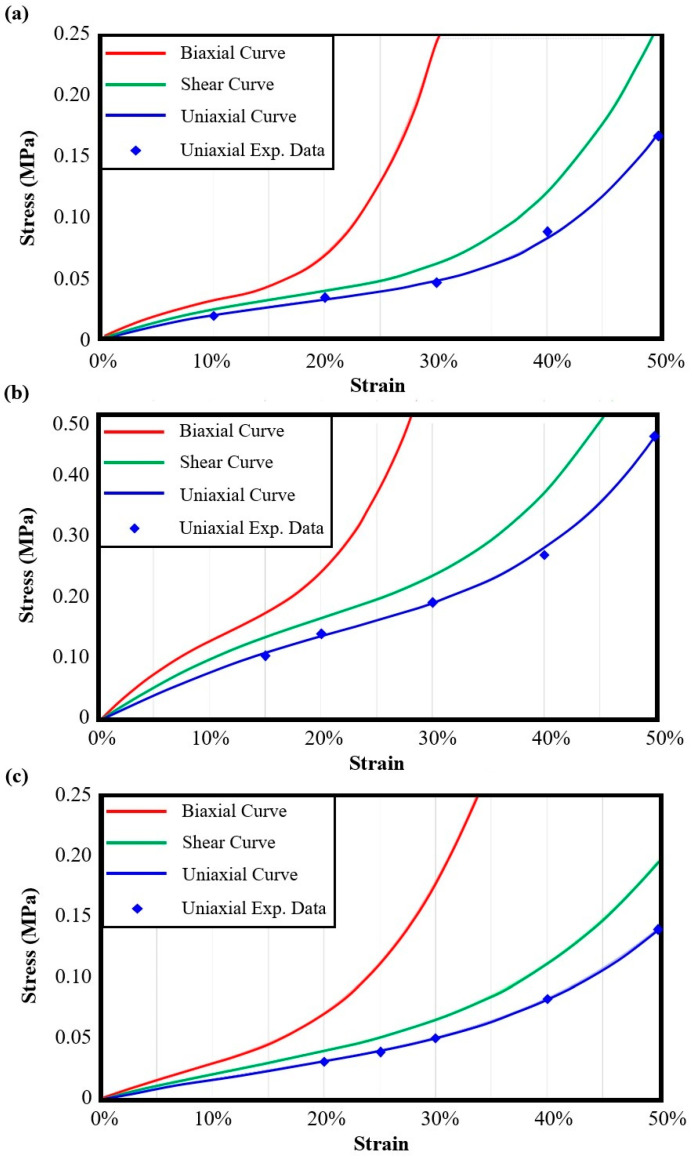
Curve fitting process for the 3rd-order Yeoh hyperelastic models for the (**a**) RE, (**b**) SM3 and (**c**) HM3 auxetic structures.

Applying the constant values that are listed in Table 6, the stress–strain curve of the desired Yeoh models can be extracted for uniaxial, shear and biaxial loading. The corresponding stress–strain curves for each examined auxetic material are presented in Figure 8, along with indicative experimental data from uniaxial compressive loading to assess the curve-fitting process. According to the data presented in Figure 8, the developed third-order Yeoh models properly fitted the experimental data with only negligible deviations, maintaining the R above 0.98.

**Figure 9:**
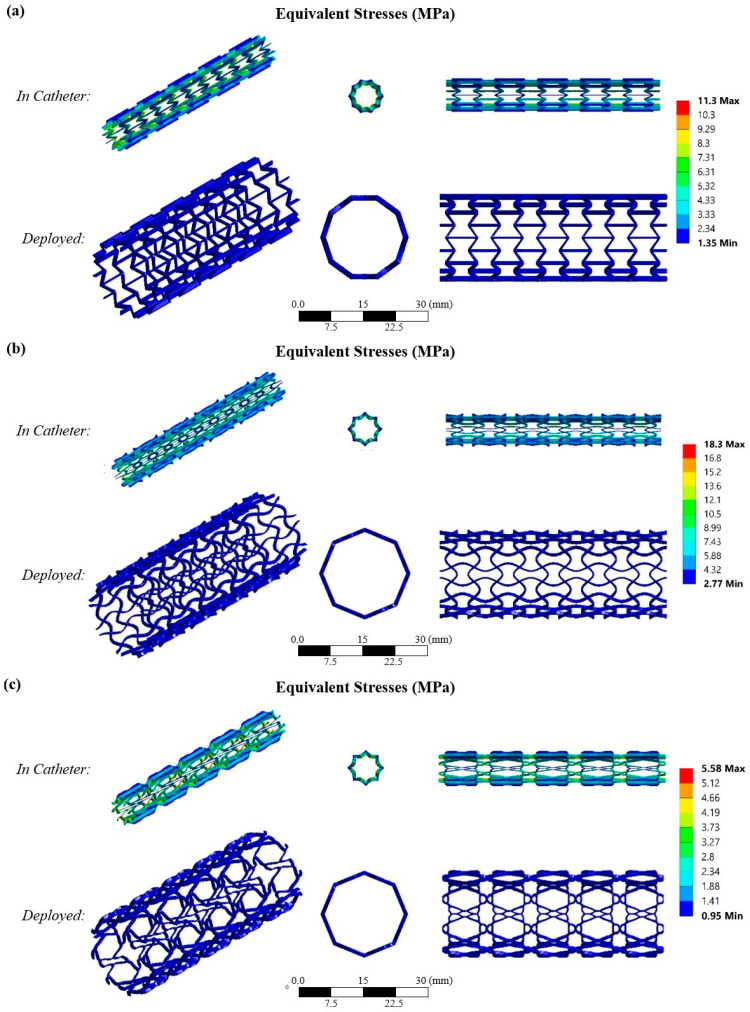
Equivalent stress contour for the developed stents with the (**a**) RE, (**b**) SM3 and (**c**) HM3 auxetic structures, in the deployed phase (unstressed) and inside the catheter (compressed).

Having set the proper non-linear hyperelastic FE model, the FEAs were then conducted. Figure 9 shows the equivalent stress contours for the two examined phases of the stent, namely when it is deployed (strain 0%) and when it is inside the catheter (strain ≈ 70%), for all three developed stents. Due to the fact that the loading condition is the applied radial displacement of the stent, the extracted stresses revealed which structure can withstand higher loads in order to achieve the desired diameter reduction. In this context, the results show that all the structures achieved the targeted diameter without exceeding the material’s yield point. In detail, the SM3 structure revealed the most uniform stress distribution, achieving maximum stress of around 18 MPa. The RE structure also achieved a good stress distribution, with maximum stress of around 11 MPa. On the other hand, HM3 showed specific regions that were more vulnerable than others, accumulating stress, resulting in maximum stress of 5.5 MPa. Therefore, SM3 can withstand the highest loads, as achieving the targeted diameter requires applying increased forces. Following this, the RE structure demonstrates slightly lower load resistance, with the HM3 structure exhibiting the least capacity to handle loads. Then, the RE structure was followed, and finally, the HM3 structure. Hence, the most suitable candidate for stent application is the SM3 auxetic material due to the fact that it has the necessary compressibility in order to modify its shape but also to withstand the highest loads in order to withstand the load from the aortic walls.

## 4. Conclusions

This paper presents the development and evaluation of three different auxetic architected materials in order to examine their potential in 4D-printed stent applications. Specifically, three novel auxetic materials were designed and test specimens were fabricated using 3D printing with PCL as the construction material. Their mechanical behavior was subsequently evaluated through uniaxial quasi-static tests. Based on the experimental results, non-linear hyperelastic FE models were developed. In the final phase, these models were utilized to evaluate the strength, feasibility and functionality of applying and integrating these auxetic materials in the actual stent’s geometry. Computational analysis revealed that the developed auxetic materials are suitable for 4D-printed stent applications due to their ability to shape-shift without compromising their overall structural integrity. Among the materials, the SM3 structure revealed the best performance in terms of the strength and load handling, while maintaining the necessary compressibility (diameter reduction) for insertion into a standard catheter during the stent procedure. While the RE and HM3 structures also passed the compressibility tests, their strength was deemed insufficient to withstand arterial wall loads. Thus, SM3 emerges as the most promising candidate for 4D-printed stent applications. When comparing the developed stent designs to conventional stents and existing 4D-printed structures reported in the literature, the SM3 auxetic material on PCL 3D-printed stents demonstrates several advantages. Unlike traditional stents, which often rely on linear elastic materials and uniform geometries, the SM3 structure leverages its auxetic properties to achieve enhanced compressibility and adaptability to complex arterial geometries without compromising its strength. Furthermore, compared to other 4D-printed stents, the SM3 design stands out due to its superior load-handling capabilities and resilience during shape transformation. The customization offered by 3D printing allows for patient-specific designs, which can be tailored to individual anatomical requirements, enhancing the overall efficacy. Additionally, the use of PCL as a construction material not only enables enhanced bioactivity and biodegradability but also eliminates the need for secondary operations to remove the stent in cases where it is only temporarily required, such as in pediatric patients with congenital heart defects requiring temporary vascular support. It is also noteworthy that the majority of existing 4D-printed stents are constructed from metals, which lack the biodegradability and flexibility provided by PCL. These comparisons underscore the potential of the SM3 structure to offer improved performance in stent applications. However, further research is required to assess its durability under cyclic pulsatile loads (e.g., heartbeats), its impact on the blood flow within the artery, and its biodegradability during deployment in the human body.

## Figures and Tables

**Table 1:** Wall thickness values for the auxetic material specimens at each relative density.

Auxetic Architected Materials	Wall Thickness (mm)	Relative Density
RE	0.4	15%
	0.8	30%
	1.2	45%
SM3	0.4	20%
	0.8	35%
	1.2	50%
HM3	0.4	15%
	0.8	30%
	1.2	45%

**Table 2:** Three-dimensional printing parameters of the PCL material.

3D Printing Parameters	
Nozzle diameter	0.4 mm
Cold Extrusion	Enabled (M302)
Extrusion temperature	90 °C
Build platform temperature	40 °C
Layer height	0.2 mm
Infill percentage	Solid
Print speed	40 mm/s
Platform adhesion	Brim (5 mm)

**Table 3:** Experimental mechanical properties of the PCL material based on a previous study [40].

Material Properties	
Density	1.2 g/cm^3^
Poisson’s ratio	0.45
Elastic modulus	345 MPa
Yield strength	14 MPa
Ultimate tensile strength	17 MPa
Compressive strength	25 MPa
Elongation at break	460%

**Table 4:** Acquired experimental values of the main effective mechanical properties for the examined auxetic structures.

Auxetic Architected Materials	Elastic Modulus [MPa]	Yield Stress [MPa]	UTS [MPa]	Compressive Strength [MPa]	Relative Density
RE	1.9 ± 0.1	0.49 ± 0.05	0.62 ± 0.05	0.16 ± 0.05	15%
	3.6 ± 0.1	1.25 ± 0.1	1.56 ± 0.1	0.55 ± 0.05	30%
	5.8 ± 0.3	1.61 ± 0.1	2.01 ± 0.1	1.26 ± 0.1	45%
SM3	6.5 ± 0.3	1.19 ± 0.1	1.49 ± 0.1	0.44 ± 0.05	20%
	12 ± 0.5	2.16 ± 0.1	2.68 ± 0.1	1.24 ± 0.1	35%
	19 ± 0.5	3.49 ± 0.2	4.37 ± 0.2	2.38 ± 0.1	50%
HM3	1.2 ± 0.1	0.49 ± 0.05	0.53 ± 0.05	0.13 ± 0.05	15%
	3.1 ± 0.1	1.28 ± 0.1	1.59 ± 0.1	0.67 ± 0.05	30%
	10 ± 0.5	3.43 ± 0.2	4.28 ± 0.2	1.24 ± 0.1	45%

**Table 5:** C and n values of the scaling laws for the main effective mechanical properties.

Auxetic Architected Materials	Elastic Modulus [MPa]		Yield Stress [MPa]		UTS [MPa]		Compressive Strength [MPa]	
	C_EM_	n	C_YS_	n	C_UTS_	n	C_CS_	n
RE	0.037	1.006	0.248	0.960	0.248	0.959	0.245	1.983
SM3	0.126	1.2	0.563	1.227	0.563	1.227	0.340	1.834
HM3	0.255	2.729	1.231	2.077	1.229	2.075	0.199	1.726

**Table 6:** Constant values for the developed 3rd-order Yeoh models.

Auxetic Architected Materials	3rd-Order Yeoh Model Constants [MPa]		
	C_1_	C_2_	C_3_
RE	0.0356	−0.0352	0.0818
SM3	0.1374	−0.0772	0.1537
HM3	0.0265	0.0022	0.0304

## Data Availability

The original contributions presented in this study are included in the article. Further inquiries can be directed to the corresponding authors.
